# Recent Progress in Understanding the Action of Natural Compounds at Novel Therapeutic Drug Targets for the Treatment of Liver Cancer

**DOI:** 10.3389/fonc.2021.795548

**Published:** 2022-01-26

**Authors:** Yannan Zheng, Wenhui Zhang, Lin Xu, Hua Zhou, Man Yuan, Hongxi Xu

**Affiliations:** ^1^ School of Pharmacy, Shanghai University of Traditional Chinese Medicine, Shanghai, China; ^2^ Engineering Research Center of Shanghai Colleges for Traditional Chinese Medicine (TCM) New Drug Discovery, Shanghai, China; ^3^ School of Chinese Medicine, Li Ka Shing Faculty of Medicine, The University of Hong Kong, Hong Kong, Hong Kong SAR, China; ^4^ Shuguang Hospital, Shanghai University of Traditional Chinese Medicine, Shanghai, China

**Keywords:** natural compound, HCC, liver cancer, signaling pathway, target

## Abstract

Liver cancer is the third most common cause of cancer-related death following lung and stomach cancers. As a highly lethal disease, liver cancer is diagnosed frequently in less developed countries. Natural compounds extracted from herbs, animals and natural materials have been adopted by traditional Chinese medicine (TCM) practices and reported to be effective in the development of new medications for the treatment of diseases. It is important to focus on the mechanisms of action of natural compounds against hepatocellular carcinoma (HCC), particularly in terms of cell cycle regulation, apoptosis induction, autophagy mediation and cell migration and invasion. In this review, we characterize novel representative natural compounds according to their pharmacologic effects based on recently published studies. The aim of this review is to summarize and explore novel therapeutic drug targets of natural compounds, which could accelerate the discovery of new anticancer drugs.

## Introduction

### Liver Cancer

Liver cancer is among the top five cancers in both incidence and mortality across all ages and sexes (1, 2) and is the third-leading cause of cancer-related death worldwide, following lung and stomach cancers (3–5). Liver cancer comprises diverse primary hepatic tumors (6). As a highly lethal disease, liver cancer is diagnosed frequently in less developed countries (7). In 2012, more than 782,500 new liver cancer cases were diagnosed, and more than 745,000 liver cancer-related deaths were recorded globally; of these, half of the total numbers of cases and deaths occurred in China (2, 7–9). Furthermore, the incidence of liver cancer is rising in many countries, including China, the United States and some European countries (2, 10). However, recent research indicates that the highest rates of liver cancer-related death are found in East and Southeast Asia (2).

### Hepatocellular Carcinoma

Hepatocellular carcinoma (HCC) is the most common histological subtype of primary liver cancer, accounting for 70% to 85% of the total liver cancer burden worldwide (11). Although surgical and medical treatments are available, drugs can cause severe side effects, and surgery offers limited treatment, especially for patients with advanced HCC (12). Sorafenib (Nexavar, Bayer Healthcare Pharmaceuticals, Onyx Pharmaceuticals) is one of several anti-HCC drugs and can prolong overall survival and progression-free survival by almost 3 months in patients with advanced HCC (12). However, its therapeutic potential is limited by its adverse effects, drug resistance and high cost (13). Additionally, there is still a high likelihood of progressive disease outside of the treated volumes following radiotherapy (RT) (14). Therefore, it is necessary to find a novel effective therapeutic treatment that can be used to control the progression and prognosis of HCC.

### Natural Compounds

Natural compounds extracted from herbs, animals or natural materials have been reported as effective compounds in the development of new medications for the treatment of diseases (15). More than 60% of drugs used to treat cancers originate from natural products (16, 17). For example, oblogifolin C (OC), a compound extracted from *Garcinia yunnanensis* Hu, has been reported to be effective against cancer (18). In recent years, Chinese herbal extracts or natural compounds isolated from traditional Chinese medicines (TCMs) have been used to treat patients with liver cancer (19, 20). For instance, curcumin is used for the treatment of HCC due to its multiple pharmacologic effects against HCC (13, 21). Recent evidence indicates that most anticancer drugs that have been applied for the treatment of HCC have serious side effects (15), whereas natural compounds with effective anticancer activities tend to have fewer side effects (22, 23), although their pharmacological mechanisms of action in combating HCC are complex and require further elucidation (24).

### Pharmacological Mechanism and Targets

Over the past twenty years, compelling functional studies have shown that cell death is a natural barrier to cancer development (25). It is widely known that apoptosis is triggered in response to cell death signaling (25, 26), and recent reports further indicate that autophagy and necroptosis can mediate cell death (27). Recent evidence shows that natural compounds can target one or multiple signaling pathways to induce apoptosis (28–30). For example, psoralen, a major active component of *Psoralea corylifolia*, induces apoptosis in liver cancer cells (31). Arresting the cell cycle is another common way to control the growth of cancer cells. At the molecular level, liver cancer is characterized by disruptions in cell cycle regulatory processes *via* various pathologic mechanisms (32). It is important to focus on the mechanisms by which natural compounds affect the cell cycle in HCC. In addition, some natural compounds have been observed to inhibit the migratory and invasion abilities of cancer cells. For example, Licochalcone A, a compound isolated from the root of *Glycyrrhiza inflata*, suppresses the migration and invasion of HCC *via* the downregulation of MKK4/JNK (33).

**Figure 1:**
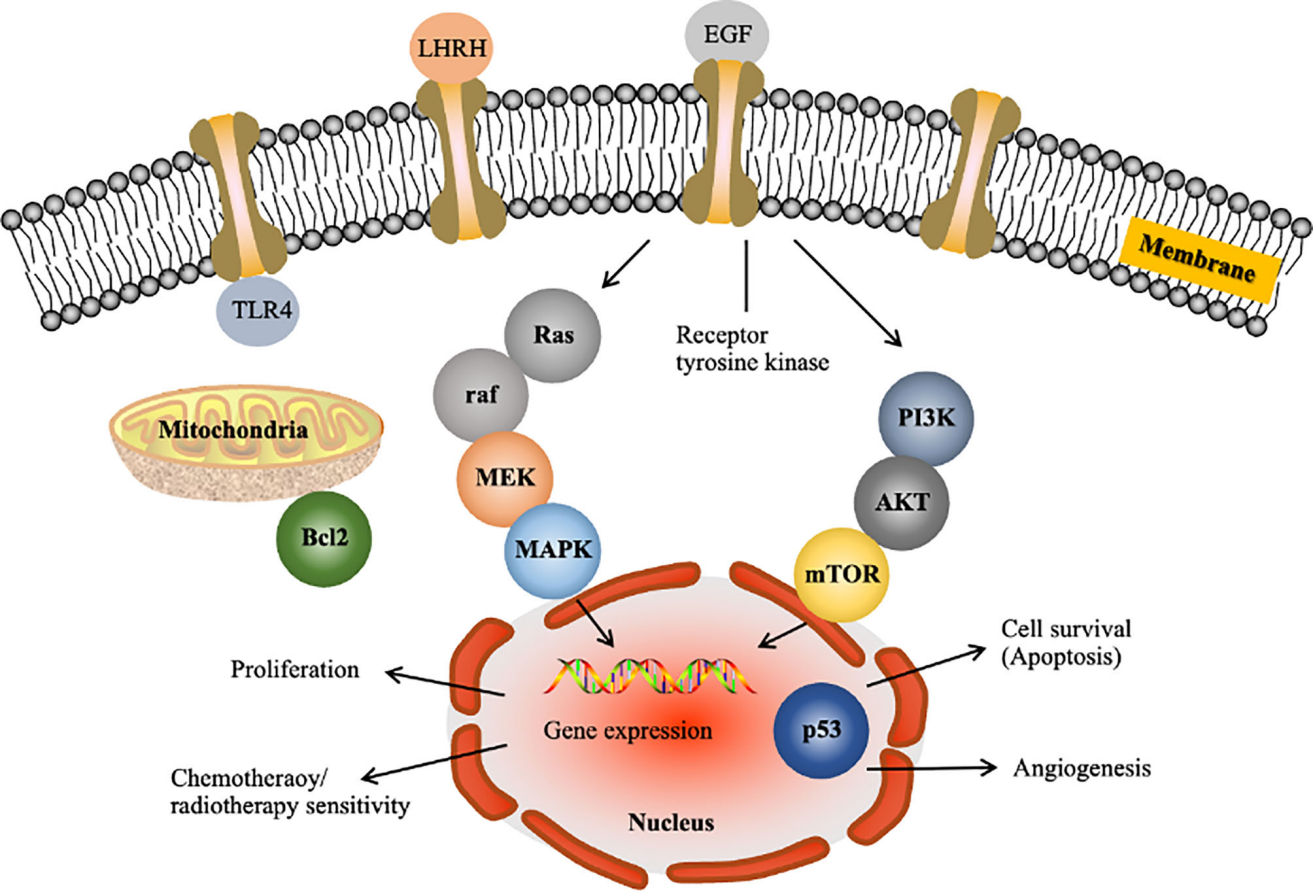
Examples of molecular targets in tumor cells for liver cancer drug development. LHRH, luteinizing hormone releasing hormone; EGF, epidermal growth factor; TLR4, Toll-like receptor 4; MAPK, mitogen-activated protein kinase. The figure shows the following two major pathways implicated in HCC: the Raf/MEK/Erk pathway, which is the prototypical MAPK cascade (35), and the PI3K/Akt/mTOR pathway (36).

Over the past few decades, the methods used to identify anticancer targets have fundamentally changed (34). Scientists are currently able to identify genes at the molecular level and investigate the pharmacological mechanisms underlying the anticancer action of a drug. **Figure 1** shows some gene products known to affect cell cycle progression, apoptosis, migration, invasion and angiogenesis. Based on the literature, the major pathways implicated in HCC are the RAF/MEK/ERK (**Figure 1**), phosphatidylinositol-3 kinase (PI3K)/AKT/mammalian target of rapamycin (mTOR) (**Figure 1**), WNT/β-catenin, insulin-like growth factor, hepatocyte growth factor/c-MET and growth factor-regulated angiogenic signaling pathways (37). These pathways may be targeted to reverse, delay or prevent tumorigenesis. In earlier studies, the approaches used to inhibit Ras function failed due to insufficient knowledge regarding its downstream targets Raf and MEK (38). Thus, it is necessary to understand extended signalling pathways to identify realistic targets.

### The Aim of This Review

In this review, we aim to characterize novel representative natural compounds from recently published papers according to their pharmacologic effects on apoptosis, migration, invasion and cell cycle progression. In addition, we review several anti-HCC natural compounds and the major molecular signalling pathways they target in HCC. Studies investigating these natural compounds were identified by searching the literature for English language papers in the PubMed, UCL Library and Web of Science and Medline databases.

## The Pharmacology of Natural Compounds in HCC Therapy

### Cell Cycle

Cell cycle dysregulation is one of the most important characteristics of liver cancer. Cell cycle progression is mainly mediated by cyclin-dependent kinases (CDKs) (32). After a CDK binds its regulatory subunit, cyclin, and undergoes site-specific phosphorylation, it is activated and allows the cell to enter a phase of the cell cycle (39). During different phases of the cell cycle, the expression of cyclins is restricted by ubiquitination and other transcriptional regulators of cyclin genes, while the expression of CDKs is stable and constitutive (40). There are five main phases of the cell cycle as follows: G0 (quiescent state); G1, S and G2 phases (all of which are considered interphases); and M phase (cell division stage); the transitions between the phases require different cyclin-CDK complexes. During the progression from the G1 to S phase, cyclin D/E binds and activates CDK2/4/6; subsequently, CDK2/4/6 phosphorylates retinoblastoma protein (pRB) (39). Hyperphosphorylated pRB can dissociate complexes of E2Fs, allowing the cell to enter the S phase (41). The transition from the G2 to M phase is controlled by the cyclin B–CDK1 complex. CDK1 is rendered inactive *via* the phosphorylation by Wee1 and Myt1. The removal of the inhibitory phosphate of CDK1 by the phosphatase CDC25C allows entry into mitosis (42). Therefore, many natural compounds inhibit tumor proliferation by targeting either the activity of CDKs or the expression of cyclins.

Cell cycle checkpoints are surveillance mechanisms in eukaryotic cells that allow the repair of cellular damage in response to stress (43). Regulators usually act on different checkpoints, including the G1-S, G2 and mitotic spindle checkpoints. At the G1-S checkpoint, the two families of CDK inhibitors are INK4 and Cip/Kip. The INK4 family (including p16, p15, p18, and p19) negatively regulates only CDK4/6-cyclin D complexes at the G1 checkpoint, while the Cip/Kip family (including p21, p27, and p57) can inhibit all CDK-cyclin complexes (32). At the G2 checkpoint, human checkpoint kinase 2 (Chk2) and Chk1, which are activated by ATM (ataxia telangiectasia mutated) and ATR (ATM and Rad3-related) signalling, respectively, phosphorylate CDC25C, which exposes the binding site on CDC25 for the 14-3-3 protein. This interaction results in the inhibition of CDK1. The mitotic spindle checkpoint is disrupted by kinetochore-associated proteins, including MAD2, BUBR1, BUB1 and BUB3 proteins (39). Under normal conditions, there is only transient arrest between the phases. However, with the application of drugs targeting specific regulators, the cell cycle could be arrested at a fixed checkpoint, followed by apoptosis.

In this review, the natural compounds discussed are mainly categorized based on when they arrest the cells in the cell cycle, which can be measured *via* flow cytometry. The largest group of compounds arrests HCC at the G2/M phase. Asparanin A (44), chalcone flavokawain B (FKB) (45), and curcumin (46) induce G2/M phase arrest by regulating the levels of cyclins, CDKs, CDK inhibitors and related proteins. For example, asparanin A (44), wentilactone B (WB) (47) and curcumin (46) upregulate the p21-mediated inhibition of CDK-cyclin complex formation, and WB (47) and FKB (45) downregulate CDC25C expression, resulting in the inhibition of CDK1. Hellebrigenin increases the levels of p-ATM, which increases the level of p-Chk2 and, consequently, reduces the level of CDC25C (48, 49). Sun et al. found that isocorydine could upregulate Chk1 expression to induce G2/M arrest *via* CDC25C activity (50). Furthermore, these natural compounds affect upstream regulators to arrest tumor cells *via* different pathways, including the PIK3/Akt (45–48), p38 mitogen-activated protein kinase (MAPK) (46, 51), p53 (46, 47, 52–54), Ras/Raf/ERK (47) and NF-κB pathways (53). For example, WB (47) increases Raf/ERK signalling related to cell arrest and could bind GTP-Ras, resulting in downstream Raf signalling.

The second group of compounds arrests cells in the S phase by regulating the cdk/cyclin expression levels, including hispolon *via* the ERK pathway (55), *Chrysanthemum indicum* extract (56), *Mirabilis himalaica* extract (57) and Juglanthraquinone C (58) regulate the cell cycle *via* p21 mediation.

The third group of compounds arrests cells in the G1 phase and includes isosuillin (via p-Rb/E2F-1 regulation) (59), resveratrol and tanshinone II-A (via the p53 pathway), and silibinin (via p-Rb and p21 regulation) (46). However, two drugs were not mentioned in these three groups because they can arrest cells in multiple phases. 2’-Epi-2’-O-acetylthevetin B (GHSC-74) (60) induces S and G2/M phase arrest *via* the downregulation of the CDK1 and cyclin B1 protein levels, while naringenin (61) induces G1 and G2/M phase arrest *via* the p53 pathway.

### Apoptosis

Apoptosis, a type of programmed cell death, is the most common form of cell death and is a spontaneous barrier to cancer development (27). The following three different pathways initiate apoptosis: the extrinsic or cell-surface death receptor pathway, intrinsic mitochondrial pathway and intrinsic endoplasmic reticulum (ER) pathway. Caspase, a latent protease during homeostasis, plays an important role in regulating apoptosis because upstream and downstream caspases function as initiators and effectors, respectively. Caspase-8, caspase-9 and caspase-12 are upstream caspases in the extrinsic, intrinsic and intrinsic ER pathways, respectively, and converge on caspase-3, whose activation results in nuclear apoptosis (28). The cascade of proteolysis leading to the final disassembly is followed by nuclear fragmentation induced by downstream caspases (27). Several natural compounds have been shown to increase the expression of caspases to promote apoptosis in HCC. For example, GHSC-74 (60) and dentatin (62) increased the levels of caspase-9 and caspase-3, which affect the intrinsic pathway. Furthermore, asparanin A (44) and isosuillin (59) are involved in the intrinsic and extrinsic pathways because of their ability to regulate caspase-3, -8, and -9 expression. FKB (45) increased not only caspase-3 and 9 expression but also procaspase-8 and -12 activity, implying that FKB can influence the activity of all three apoptosis pathways. The cleavage of poly (ADP ribose) polymerase (PARP) caused by caspase-3 is also a marker used to measure apoptosis (50). Isocorydine and ergone increase the levels of cleaved PARP, providing further evidence of caspase-3 activation (50, 63).

The extrinsic pathway is initiated by the binding of a ligand to a death receptor, and the most well-known death receptors include CD95, TRAIL-R1/R2 and TNFR-1; these receptors contain a death domain (DD) (64), and the common ligands are Fas ligand (FasL) and TNF-related apoptosis-inducing ligand (TRAIL) (65). Signalling from proteins containing DDs is responsible for the recruitment and assembly of the death-inducing signalling complex (DISC), which comprises a Fas-associated death domain (FADD) or TNF receptor-associated death domain (TRADD) and procaspase-8/-10 (28). Isosullin (59) increases FADD expression, resulting in the activation of the extrinsic pathway. Neriifolin (66) was also observed to increase the levels of Fas and FasL, and the gene expression of the TNF ligand and TNFR was elevated upon treatment with osthole (54).

The intrinsic ER pathway, which is a mitochondria-independent pathway, is a unique apoptosis pathway activated in response to ER stress and involves disruption of ER function due to cellular stressors, such as oxidative stress and glycosylation inhibition (28). Alterations in ER homeostasis trigger specific signalling pathways; for example, the unfolded protein response (UPR) can induce the disassociation of the adaptor protein TNF receptor-associated factor 2 (TRAF2) from procaspase-12, leading to caspase-dependent apoptosis (67). 6-Shogaol (46) is an example of a natural compound that targets this pathway. One group found that 6-shogaol regulated ER signalling-dependent apoptosis *via* the PKR-like ER kinase (PERK)/elF2a pathway. PERK, as a UPR sensor, is activated upon the accumulation of misfolded proteins, which, in turn, leads to the phosphorylation of its downstream target elF2α (68). Phosphorylated elF2α prevents misfolded protein accumulation; therefore, decreased levels of elF2α promote apoptosis (69). 6-Shogaol not only induced the upregulation of PERK and downregulation of elF2α but also elevated the level of caspase-3, implying that HCC apoptosis induced by 6-shogaol depends on the intrinsic ER pathway.

The intrinsic pathway, which is widely acknowledged as the primary form of apoptosis that mitigates cancer development (27), involves the regulation of mitochondrial outer membrane permeabilization (MOMP) by Bcl-2 family proteins. The proapoptotic members of the Bcl-2 family include Bax, Bak, Bad, Bcl-X_s_, Bid, Bik, Bim and Hrk, and the antiapoptotic members include Bcl-2, Bcl-X_L_, Bcl-W, Bfl-1, and Mcl-1. The latter group acts as inhibitors of apoptosis mainly by binding and restraining proapoptotic proteins (e.g., Bax and Bak) (28). Thus, the ratio of Bax/Bcl-2 has been considered an important marker of the activation of the intrinsic pathway. The compounds arctigenin (52), celastrol (70) and phoyunbene B (53) all increase the ratio of Bax/Bcl-2, indicating that these drugs play proapoptotic roles in the caspase-8 pathway. Two research groups analyzed this ratio with the drug osthole (54, 71) but obtained conflicting results; Zhang et al. found no large difference, while Chao et al. reported that the ratio increased. There discrepancies were likely due to the investigation of different types of expression as the former study examined expression at the gene level, while the latter study measured protein levels. Mcl-1 is an effector downstream of many important pathways involved in the response to cellular damage (72); thus, Mcl-1 expression has been investigated in many studies. Silibinin (49) and ψ-Bufarenogin (73) induced the downregulation of Mcl-1 protein and mRNA expression. Moreover, the expression of Bid, as a linker between death receptors and the mitochondrial apoptosis pathway (74), was examined in response to treatment with Momordica charantia lectin (MCL) (75), and the results showed that the expression of truncated bid was increased, leading to apoptosis. Bax and Bak, which are located on the outer mitochondrial membrane, promote the release of cytochrome c, which is the most important proapoptotic signalling protein (27). Cytochrome c binds the adaptor protein apoptotic protease-activating factor 1 (Apaf-1) within the caspase-9 complex, resulting in the activation of caspase-3 (72). The level of cytochrome c in the cytosol was increased by many compounds, such as furanodiene (51), 2-methoxyjuglone (76) and oleanolic acid (53). Li et al. (56) found that the expression of both Apaf-1 and cytochrome c was increased.

These proteins are not the only regulators of the three apoptosis pathways. In addition, MAPK pathways, including the ERK, JNK and p38 pathways, have a profound influence on apoptosis (77). The level of phosphorylated ERK was increased in response to treatment with furanodiene (51) and hispolon (55), indicating that both drugs induced apoptosis, at least partially, *via* the ERK pathway. Furthermore, Longikaurin A (78) increased the level of phosphorylated JNK and its downstream effector c-jun, leading to apoptosis. The level of phosphorylated JNK also increased upon treatment with WB (47), leading to elevated Bad levels; thus, WB likely induced apoptosis *via* the Ras/JNK pathway. Increased levels of phosphorylated p38 were observed upon treatment with protocatechuic acid (PCA) (79) and furanodiene (51), indicating that these two drugs induce apoptosis by promoting p38 MAPK signalling. In contrast, FKB (45) could inhibit p38 but also induce apoptosis likely because although p38 may be involved in apoptosis, inhibiting p38 decreases the level of Bcl-2, which is an anti-apoptotic protein.

p53 is an important transcription factor that regulates the expression of apoptosis-related genes in response to cellular stresses. As a tumor suppressor protein, it tightly regulates cell growth by promoting apoptosis and DNA repair under stressful conditions (80). More than 50% cancer patients harbor somatic mutations in p53 genes, so it is not surprised that p53 has been an attractive target for cancer therapy. Strategies have been developed to target p53, including gene therapy to restore p53 function and inhibition of p53-MDM2 interactions (81). p53 regulates distinct aging hallmarks, which is closely related to cancer and aging therapeutics. MDM2 protein is a core negative regulator of p53 and maintains low levels of p53 in normal cells (82). Direct regulation of the p53-MDM2 axis is associated with elevated p53 activity, both in early aging or with delayed-onset aging (83). Many drugs, such as isosuillin (59), psoralen (31) and berbamine (84), induce apoptosis *via* the p53-dependent pathway. NF-κB is another target for regulating apoptosis. Dentatin (62) could induce the downregulation of NF-κB, leading to decreased levels of inhibitor of apoptosis proteins (IAPs), which directly suppress caspase activation. Therefore, the downregulation of NF-κB could promote apoptosis in HCC. However, NF-κB has been shown to promote the expression of antiapoptotic proteins, such as Bcl-2 and Bcl-xL (85). Hydroxytyrosol inhibited the activity of NF-κB, leading to the downregulation of these genes (86). Moreover, the PI3K/Akt pathway, a cell proliferation-related pathway, was inhibited by ψ-bufarenogin, leading to decreased levels of the antiapoptotic protein Mcl-1, which is also involved in the induction of apoptosis.

### Autophagy

Autophagy is a basic catabolic process that degrades damaged cellular components and recycles normal organelles to maintain cellular homeostasis (87). Interestingly, autophagy plays dual roles in cell survival and cell death during cancer development, which is often related to its complex relationship with apoptotic machinery (88). For example, although platycodin D (PD) (89) induces cell death mainly *via* autophagy, it can trigger a cytoprotective mechanism in HepG2 cells. However, autophagy plays a more critical role in the cell death pathway in developing tumors and remains a therapeutic aim for treating HCC. LC3 is a basic marker of autophagosomes, and PD (89) and MCL (75) can induce higher levels of LC3-II. As the *Atg-5* and *Atg-7* genes are related to autophagosome formation, the deletion of the *Atg-5* gene in mice can cause the development of multiple benign tumors in the liver, and the liver-specific deletion of Atg-7 in mice also induces tumor production. Therefore, the expression level of these two genes is a marker of autophagy in tumors. Tetrandrine (90) upregulated the expression of Atg-7, while oroxylin A (91) induced the overexpression of *Atg-5* and *Atg-7*; both drugs induced cell death *via* autophagy. There are many important targets in the autophagic pathway as follows: Beclin-1, p53 and mammalian target of rapamycin complex 1 (mTORC1) (65). Beclin-1 is an important autophagic regulator that can interact with members of the Bcl-2 family (92) as it contains a BH3 domain. Beclin-1 disassociates from Bcl-2 family proteins upon interaction with stress sensor-coupled BH3 proteins and induces autophagy and apoptosis by the release of proapoptotic proteins (e.g., Bax and Bak) (27). Oroxylin A (91) and berberine (93) upregulate the expression of beclin-1, resulting in the induction of autophagy and apoptosis. p53 can trigger cell death when located in the nucleus but inhibits autophagy when located in the cytoplasm (94). Cytoplasmic p53 activates the downstream effector AMPK, which phosphorylates TSC2, which, in turn, inhibits mTOR signalling and prevents the cell from undergoing apoptosis (95). Allicin induced autophagy by decreasing the levels of cytoplasmic p53. Furthermore, PD (89) and tetrandrine (90) trigger autophagy *via* the ERK pathway, and oroxylin A (91) induces HCC death by inhibiting the PI3K/mTOR pathway. These results show that the downregulation of PI3K leads to decreased levels of phosphorylated Akt. Moreover, the expression of mTOR and PTEN, two tumor suppressor proteins, was elevated. Therefore, oroxylin A induced autophagy *via* the PI3K-PTEN-Akt-mTOR signalling pathway. Another therapeutic target for tumor, p62, known as stress-inducible cellular protein, interacts with various signaling proteins to regulate cellular functions (96). It also serves as a classical receptor of autophagy, involved in many signal transduction pathways (97). Growth of liver tumors caused by the inhibition of autophagy can be greatly diminished by concomitant deletion of p62 or Nrf2. Interestingly, upregulation of p62 was also observed to be advantageous for cancer therapy. Through controlling DNA-damage-induced histone H2A ubiquitination in autophagy-deficient cells, p62 increases the sensitivity of cancer cells to radiation (98).

Thus, modulating cellular levels of p62 in cancer therapeutics is important.

### Invasion and Migration

Cancer cell metastasis is a complex process that requires invasion, migration, adhesion and angiogenesis (99). To initiate the metastatic process, cancer cells acquire the ability to invade membranes and disrupt intercellular interactions, which is a critical characteristic of malignant tumors (100). Subsequently, invading tumor cells dissolve the extracellular matrix (ECM), which, when occurs excessively, is a hallmark of tumor invasion and migration (27). Thus, cancer cells migrate and invade by damaging the ECM (101). Recent research has demonstrated that some natural compounds present anticancer potential in HCC by inhibiting migration and invasion. One example is PD, which is isolated from Platycodonis Radix and has been reported to effectively inhibit HepG2 cell migration and invasion in HCC cells (102).

Matrix metalloproteinases (MMPs) are proteolytic enzymes (103), are involved in ECM degradation in physiological processes (104) and can promote the migration and invasion of cancer cells (100). Among these MMPs, MMP-2 (72 kDa gelatinase) and MMP-9 (92 kDa gelatinase) are particularly crucial for cancer cell invasion and migration (105). Increasing evidence indicates that natural compounds can contribute to the inhibition of migration and invasion in HCC by downregulating MMP-2/-9. For example, portulacerebroside A, which is derived from Portulacaoleracea, could inhibit MMP-2 and MMP-9 enzyme activity to disturb the migration and invasion of human HCCLM3 cells (106).

The transcription of MMP-2/-9 genes is regulated by transcription factors, including nuclear factor κB (NF-κB) (107, 108) and activator protein-1 (AP-1) (109, 110). NF-κB is an important multisubunit transcription factor that mediates cellular responses to inflammation, cell viability and invasion (111). The natural compound gambogic acid has been shown to inhibit metastasis in SK-HEP1 cells by decreasing NF-κB expression and downregulating MMP-2 and MMP-9 *via* the regulation of the actin cytoskeleton; this occurs through the suppression of integrin β1-mediated Rho family GTPases (112). Integrins, a family of transmembrane proteins, consist of an α and a β subunit. Integrin β1 mediates the interaction between the actin cytoskeleton and ECM (113) by regulating the activity of Rho family GTPases, including Rho, Rac and Cdc42, and has been discovered to mediate invasion and migration in cancer cells in various ways (114, 115). In addition, tissue inhibitors of metalloproteinases (TIMPs) are endogenous inhibitors of MMPs (116). Controlling the balance between TIMPs and MMPs can affect the invasion and migration of cancer cells (117, 118).

**Figure 2:**
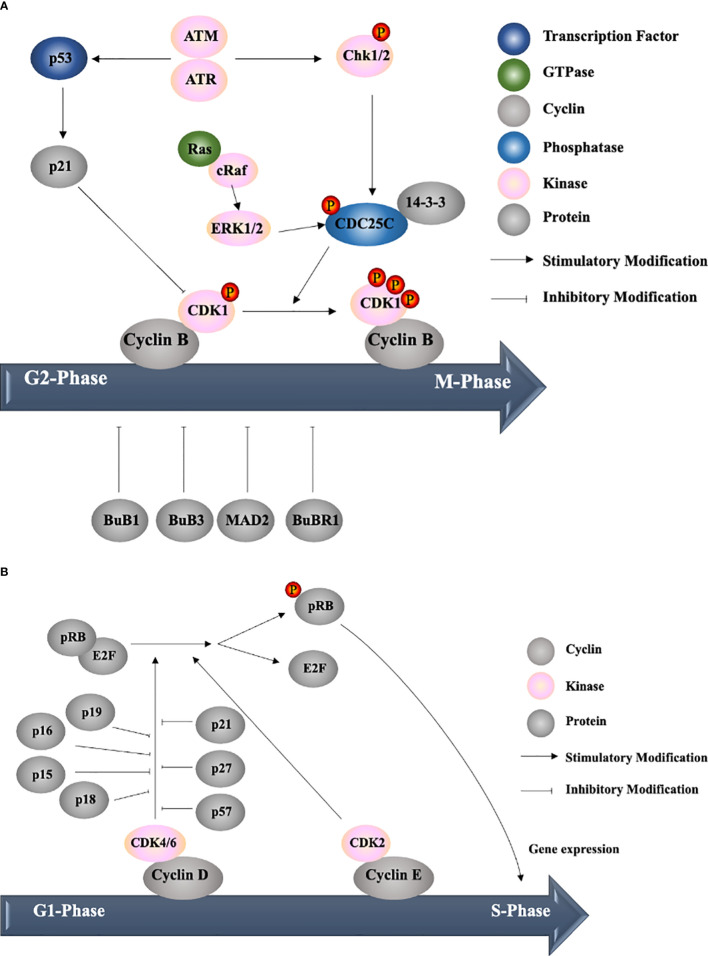
Cell cycle progression. **(A)** Example of targets of cell cycle arrest at the G2/M transition and G1/S transition in tumor cells for the development of liver cancer drugs. ATM, ataxia telangiectasia mutated; ATR, ATM and Rad3-related; Chk, human checkpoint kinase; ERK, extracellular signal-regulated kinase. **(B)** Example of targets of cell cycle arrest at the G1/S transition in tumor cells for the development of liver cancer drugs. pRB, retinoblastoma protein; CDK, cyclin-dependent kinase.

However, the processes of invasion and migration in HCC require specific intracellular signalling pathway activations, such as MAPK signalling cascades (33, 119). According to recent natural compound studies, most novel natural compounds inhibit tumor invasion and migration *via* the following three major signalling pathways to regulate MMP-2/-9: ERK1/2, p38 MAPK and PI3K/Akt (33, 120, 121). For instance, hispolon, which is isolated from the medicinal mushroom *Phellinus linteus*, decreases the activities of MMP-2/-9 by inactivating the ERK1/2 signalling pathway, reducing RhoA protein expression and inhibiting the phosphoinositide 3-kinase (PI3K)/Akt signalling pathway in SK-Hep1 cells (100). It has been reported that urokinase-plasminogen activator (uPA) leads to the inhibition of metastasis (100, 122) and that integrin-β1 mediates focal adhesion kinase (FAK), Src, and PI3K signalling (123) to downregulate downstream Akt and suppress the expression of RhoA, Cdc42, and Rac1 (**Figure 2**). These activities contribute to the inhibition of MMP-2/-9 (112) However, some natural compounds regulate MMP-2/-9 *via* the ERK-1/-2 signalling pathway, which may be activated by its upstream regulators Raf and MEK. For example, galangin, which is found in *Alpinia officinarum*, could inhibit the suppression of MMP-2/MMP-9 enzyme activity to disturb migration and invasion in human HepG2 cells *via* the protein kinase C (PKC)/ERK cascade (124). PKC isoforms are major signal transduction enzymes that respond to extracellular signals (125).

The process of tumor metastasis is complicated. Numerous studies have investigated whether natural compounds inhibit the invasion and migration of liver cancer cells *via* various signalling pathways. VEGF receptor signalling, specifically VEGFR2, can lead to the activation of the downstream kinases Src and FAK, which induce the expression of Rho-GTPases (126, 127), whose activation results in the inhibition of cell migration and invasion (128); therefore, VEGFR knockdown reduces tumor migration. For example, corosolic acid in *Actinidia chinensis* can block the VEGFR2 ATP binding pocket and mitigate the downstream Src/FAK/cdc42 signalling pathway to reduce the migration and invasion of Huh7, HepG2, and Hep3B cells *in vitro* and *in vivo* (129).

In addition, it has been reported that reactive oxygen species (ROS) induce downstream VEGFR activation (130), resulting in the accumulation of unfolded proteins in the ER lumen and subsequent ER stress (131). Accumulating evidence suggests that ER stress mediates inositol-requiring kinase 1 (IRE1) to regulate Akt expression, which could inhibit cell migration (132). Piperlongumine and bufalin reduce tumor migration by activating ER stress (132–134). A recent study investigating brucine, which is an alkaloid derived from the seeds of *Strychnos nux-vomica* Linn, indicated that brucine inhibits the migration and invasion of cancer cells by suppressing the transcriptional activity of hypoxia inducible factor 1 (HIF-1), a critical transcription factor activated during hypoxic stress (135). (-)-Epigallocatechin-3-gallate (EGCG), a compound isolated from green tea, can inhibit thrombin-induced migration and invasion *via* the ERK1/2 pathway (136), suggesting that thrombin has antimetastatic effects on liver cancer cells.

Overall, the inhibition of MMP-2/-9 and the balance between MMP-2/-9 and TIMP-1/-2 could play a critical role in liver cancer therapy. Furthermore, several natural compounds could exert their inhibitory effects on cell migration and invasion *via* various pathways.

## Discussion and Conclusion

In the battle against HCC, natural compounds have been investigated with the goal of developing new drugs with potent anticancer activities.

Thus far, several natural compounds, such as curcumin, silibinin, berberine and quercetin, have been proven to induce cell cycle arrest (**Table 1**), apoptosis (**Table 2**) and autophagy (**Table 3**) and inhibit cell metastasis (**Table 4**) in HCC *via* different pharmacologic mechanisms and signalling pathways (21). Regarding the cell cycle, some herbal compounds target the regulation of CDK and cyclin expression in different phases to prevent tumor proliferation. It can be deduced from recent research that most natural compounds regulate p21 or modify CDC25C *via* the Raf/Erk pathway to inhibit cyclin B arrest cells in the G2/M phase (**Figure 2A**). In addition, other natural compounds have been shown to inhibit CDK to arrest cells in the G1/S phase (**Figure 2B**).

**Table 1:** The targets and pathways of natural compounds in the process of arresting cell cycle progression in HCC.

Phase of cell cycle	Target	Signalling pathway	Natural compound	Chinese medicine	Reference
G2/M	cyclin A, Cdk1/4, and caspase-3/8/9	Caspase pathway	Asparanin A	Asparagus officinalis L	(44)
G2/M	cyclin B1, cdc2/25c, and Myt1	PIK3/Akt pathway	Chalcone Flavokawain B	Rhizomes of Alpinia plants	(45)
G2/M	P38 and p53	PIK3/Akt, p38 MAPK, and p53 pathways	Curcumin	Curcumalonga L	(46)
G2/M	cyclin B1 and cdc2	Ras/Raf/MAPK pathway	Wentilactone B (WB)	Aspergillus wentii EN-48	(47)
S	cdk/cyclin	ERK pathway	Hispolon	Phellinus igniarius	(55)
S	p21	p21 pathway	Chrysanthemum indicum extract	Chrysanthemum indicum	(56)
S	p21	p21 pathway	Mirabilis himalaica extract	Mirabilis himalaica	(57)
S	p21	p21 pathway	Juglanthraquinone C	Juglans mandshurica Maxim	(58)
G1	p-Rb/E2F-1		Iso-suillin		(59)
G1	p53	p53 pathway	Resveratrol	Grape	(46)
G1	p53	p53 pathway	TanshinoneII-A	Salvia miltiorrhiza root	(46)
G1	p-Rb, p21	STAT3 pathway	Silibinin	milk thistle (Silybum marianum)	(46)
S and G2/M	CDK1 and Cyclin B1	Caspase-dependent and -independent pathways	2’-epi-2’-O-Acetylthevetin B	Cerbera manghas L	(60)
G1 and G2/M	Bcl family and cytochrome c	Caspase pathway	Naringenin	Citrus fruits	(61)

**Table 2:** The targets and pathways of natural compounds in the process of inhibiting apoptosis in HCC.

Target	Signalling pathway	Natural Compound	Chinese medicine	Reference
procaspase-8/12 and caspase-9/3	p38 MAPK cascades	Chalcone Flavokawain B	rhizomes of Alpinia plants	(45)
p53 and Bcl-2 family	p53 pathway	Psoralen	Psoralea corylifolia	(31)
p53	p53 pathway	Resveratrol	Polygonumcuspidatum Sieb. etZucc.	(21)
p53	p53 pathway	Quercetin	Foods, including onions, grapes, beverages (i.e., tea, wine, and beer) and green vegetables	(21)
p53	p53 pathway	Curcumin	Curcumalonga L.	(21)
NF-κB	NF-κB pathway	Icariin Synergizes with Arsenic Trioxide	E Herba	(137)
Mcl-1 and Bcl-xL	STAT3 pathway	Silibinin	Silybum marianum	(138)
Mcl-1	PI3-K/Akt cascade	ψ-Bufarenogin	toad skin	(73)
JNK	ROS/JNK/c-Jun signalling pathways	Longikaurin A	Isodon ternifolius	(78)
Cleaved PARP and caspase-3/8/9	caspase pathway	Asparanin A	Asparagus officinalis L.	(43)
Cleaved PARP		Isocorydine	Dicranostigma leptopodum (Maxim.) Fedde)	(50)
cytochrome c	p38 and ERK MAPK pathway	Furanodiene	Curcuma wenyujin	(51)
caspase-3/9, Bcl family, and cytochrome 3	caspase pathway	2-Methoxyjuglone	Juglans cathayensis	(76)
caspase-3/9 and Bcl family	caspase pathway	Juglanthraquinone C	Juglans mandshurica Maxim	(58)
caspase-3/8/9 and Fas/FasL	p73 pathway	Wasabia japonica extract (WJE)	Wasabia japonica	(139)
caspase-3/8/9 and FADD	p53 pathway	Iso-suillin	Suillus luteus	(59)
caspase-3/8/9	p53 pathway	Berbamine	Berberis amurensis	(84)
caspase-3/8 and cleaved PARP		Longikaurin A	Isodon ternifolius	(78)
caspase-3/8, cytochrome c, and Bcl family	ERK	Hispolon	Phellinus linteus	(55)
Caspase-3, PERK/eIF2a		6-Shogaol	Zingiber Officinale	(140)
caspase-3 gene		hexane extract	Murdannia bracteata	(141)
caspase-3 and Bax	caspase-3 pathway	TanshinoneII-A	Salvia miltiotthiza Bge. (Danshen)	(21)
caspase-3	caspase pathway	GHSC-73	Cerbera manghas L.	(60)
caspase 8/9, Bid and bim	caspase pathway	Momordica Charantia lectin (MCL)	bitter gourd	(75)
capase-3/9	caspase pathway	2’-epi-2’-O-Acetylthevetin B (GHSC-74)	Cerbera manghas L.	(60)
capase-3/8/9, Fas/FasL	caspase pathway	Neriifolin	Cerbera manghas L.	(66)
capase-3/8/9, and Bcl family	p53	Ergosta-4,6,8(14),22-tetraen-3-one (ergone)	various medicinal fungi such as Polyporus umbellatus, Russula cyanoxantha, and Cordyceps sinensis.	(63)
capase-3/8/9	caspase pathway	HH-F3	Graptopetalum paraguayense	(142)
capase-3	caspase pathway	apple flavonoid-enriched fraction (AF4)	Northern Spy apples	(44)
Blc-2 family and Fas/FasL	NF-κB pathway and p53 pathway	Arctigenin	Saussurea medusa, Arctium lappaL., T. nucifera, Forsythia intermedia and tropical climbing shrub Ipomea cairica	(143)
Bcl-2,Bcl-XL and Bid		Berberine	Huanglian(Coptis chinensis Franch., Coptis deltoidea C. Y.Cheng et Hsiao, or Coptis teeta Wall.)	(21)
Bcl-2 family, IAPs, and caspase-3	NF-κB pathway	Dentatin	Clausena excavata	(62)
Bcl-2 family and caspase-3	JNK1 signalling pathway	Icaritin	Epimedium	(144)
Bcl-2 family and caspase-3		Myrtenal	cumin, pepper, mint and eucalyptus	(145)
Bcl-2 family		Celastrol	Tripterygium wilfordii	(70)
Bcl-2 family	p53 pathway	Pulsatilla saponin A	Pulsatilla chinensis regel	(52)
Bcl-2 family	p53 pathway	(E)-3-(4-hydroxy-2-methoxyphenyl)-propenoic acid 4-hydroxy-3-methoxyphenyl ester	Mirabilis himalaica	(57)
Bcl-2 family	p53 pathway	CKBM	Mangnolia officinalis	(19)
Bcl family and cytochrome c	p53	oleanolic acid	Olea europaea,Viscum album L., Aralia chinensis I.	(53)
Bcl family and cytochrome c	caspase pathway	Naringenin	citrus fruits	(61)
Bcl family	caspase pathway	phoyunbene B	Pholidota yunnanensis	(146)
Bcl family	NF-κB pathway	hydroxytyrosol (HT)	olive oil	(86)
Bcl family	caspase pathway	Methyl protodioscin	Dioscorea collettii var. hypoglauca (Dioscoreaceae)	(33)
Bcl family	caspase pathway	osthole	Cnidium monnieri (L.) Cusson	(71)
Bcl family	ERK and JNK MAPK pathways	Wentilactone B	Aspergillus wentii EN-48	(47)
apaf-1/cytochrome c/capase-9	caspase pathway	Chrysanthemum indicum extract	Chrysanthemum indicum	(56)
	JNK and p38 MAPK pathway	Protocatechuic acid (PCA)	Lonicera japonica	(79)
	PIK3/Akt pathway	Hellebrigenin	Helleborus and Kalanchoe	(48)

**Table 3:** The targets and pathways of natural compounds in the process of inhibiting autophagy in HCC.

Target	Signalling pathway	Compound	Chinese medicine	Reference
LC3-II and autophagosome	p-53 independent	Momordica Charantia lectin (MCL)	bitter gourd	(75)
LC3-II	MEK/ERK pathway	Platycodin D	Platycodonis Radix	(102)
LC3-II	p53 and PI3K/mTOR pathways	Allicin	Garlic	(95)
beclin-1	(mTOR) signalling pathway	Berberine	Coptidis rhizoma	(93)
ATG7	partially ERK	Tetrandrine	Stephaniae tetrandrae	(90)
Atg-5 and Atg-7	PI3K-PTEN-Akt-mTOR signalling pathway	oroxylin A	Scutellariae radix	(91)

**Table 4:** The targets and pathways of natural compounds in the process of inhibiting migration and invasion in HCC.

Target	Signalling pathway	Compound	Chinese medicine	Reference
VEGFR2	VEGFR2/Src/FAK	corosolic acid	Actinidia chinensis	(129)
thrombin	PAR/ERK1/2	(-)-epigallocatechin-3-gallate (EGCG)	Green tea	(136)
ROS-potentiated	hepatocyte growth factor (HGF)	Resveratrol	grape	(147)
ROS	ROS-ER-MAPKs-CHOP	Piperlongumine	longer pepper plants	(132)
Roh GTPases and MMP-2/-9	Actin Cytoskeleton and NF-jB	Gambogic Acid	Garcinia hanburyi	(112)
MMP-2/-9 and TIMP-1/-2	ERK1/2, p38MAPK, FAK/PI3K/AKT/mTOR	11-epi-Sinulariolide Acetate	Sinularia flexibilis	(148)
MMP-2/-9 and TIMP-1/-2		portulacerebroside A	Portulacaoleracea	(106)
MMP-2/-9	NF-κB	caffeic acid phenyl ester	propolis	(149)
MMP-2/-9	PI3K/Akt, ERK1/2	Hispolon	Phellinus linteus	(100)
MMP-2/-9	β1-Integrin/FAK	Marine Bromophenol Bis (2,3-Dibromo-4,5-dihydroxy-phenyl)-methane	marine algae	(150)
MMP-2/-9	PKC/ERK	Galangin	Alpinia officinarum	(124)
MMP-2、TIMP-2	binding abilities of NF-kB and AP-1	Plumbagin	Plumbago zeylanica Linn.	(151)
LC3-II, and autophagosome	p-53 independent	Momordica Charantia lectin (MCL)	bitter gourd	(75)
LC3-II	MEK/ERK pathway	Platycodin D	Platycodonis Radix	(102)
LC3-II	p53 and PI3K/mTOR pathways	Allicin	Garlic	(95)
HIF-1		Brucine	Strychnos nux-vomica Linn.,	(135)
beclin-1	(mTOR) signalling pathway	Berberine	Coptidis rhizoma	(93)
ATG7	partially ERK	Tetrandrine	Stephaniae tetrandrae	(90)
Atg-5 and Atg-7	PI3K-PTEN-Akt-mTOR signalling pathway	oroxylin A	Scutellariae radix	(91)
		Platycodin D	Platycodonis radix	(102)

**Figure 3:**
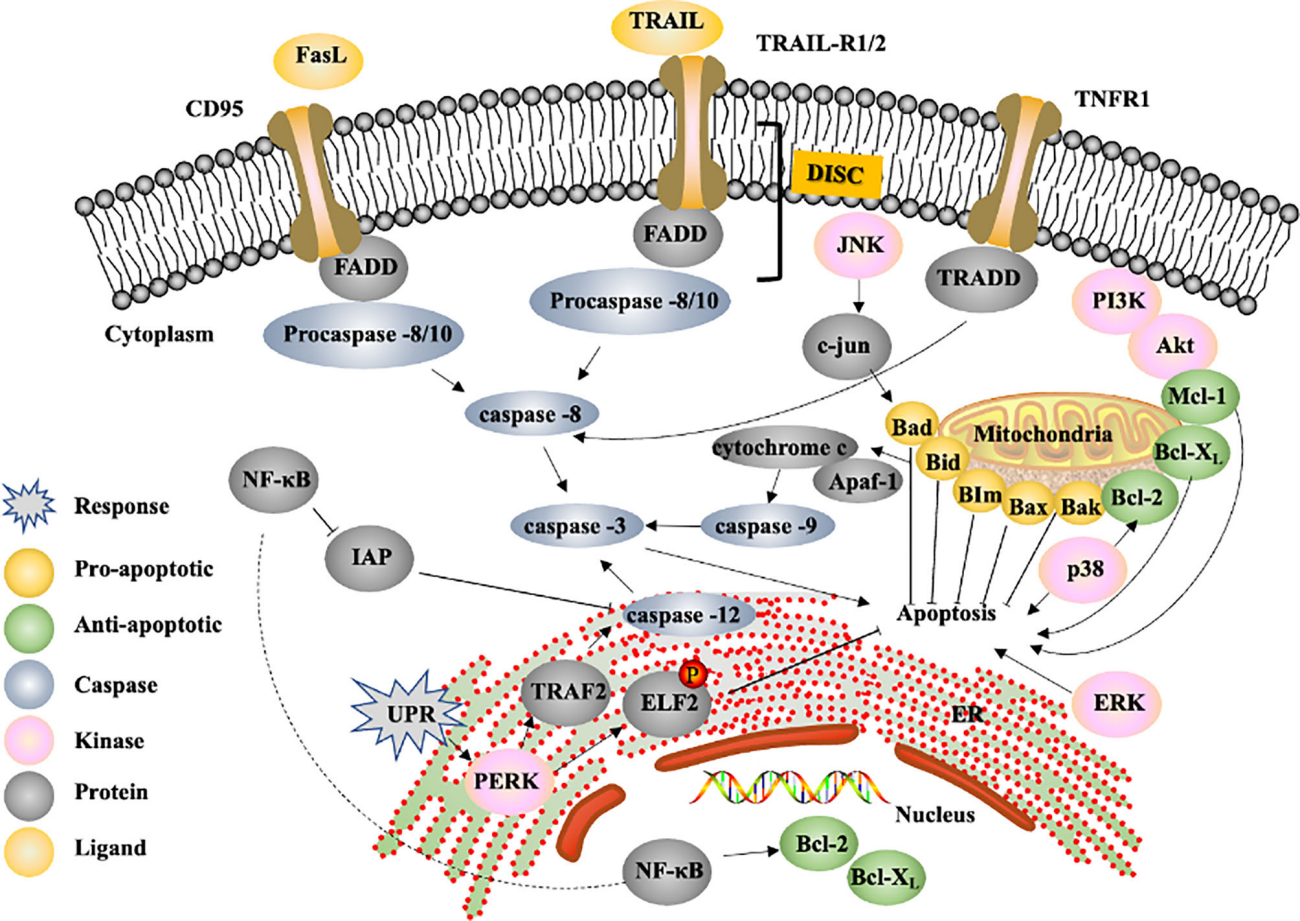
Proposed signalling pathway by which natural compounds induce apoptosis and example of targets of apoptosis in tumor cells for liver cancer drug development. FasL, Fas ligand; FADD, Fas-associated protein with death domain; TNFR1, tumor necrosis factor receptor 1; TRADD, TNFR1-associated *via* death domain; DISC, death-induced signalling complex; IAP, inhibitor of apoptosis protein; UPR, unfolded protein response; ER, endoplasmic reticulum; PERK, double-stranded RNA-activated protein kinase-like ER kinase; TRAF2, TNF receptor-associated factor 2; ELF2, eukaryotic initiation factor 2.

Apoptosis is a fundamental process that balances the development and maintenance of tissue homeostasis. In modern cancer therapy, the effective control of cancer cell apoptosis is important (152); therefore, it is critical to identify natural compounds that can accurately target the signalling intermediates in proapoptotic signalling pathways (153). The apoptosis machinery includes the following three main cascades: the extrinsic pathway, intrinsic ER pathway and intrinsic, or mitochondrial, pathway. Several natural compounds exert their activities through these pathways (**Table 2**). Recent studies have indicated that natural compounds regulate downstream caspases to induce apoptosis through the caspase pathway (**Figure 3**). In addition, the proapoptotic members of the Bcl-2 family play an important role in triggering apoptosis, and the ratio of Bax/Bcl-2 serves as an important marker of the status of the apoptosis pathway. Increasing evidence indicates that several signalling pathways are involved in inducing apoptosis, such as the MAPK, PI3K/Akt, and NF-κB pathways. ER stress could induce apoptosis *via* a mitochondria-independent mechanism (**Figure 3**). According to recent research achievements, most natural compounds, such as curcumin, have multiple targets; thus, the targets of natural compounds in the apoptosis pathway need to be clarified to better determine their pharmacologic mechanisms. The modulation of apoptosis and autophagy seems a critical aspect in the mechanism of action of natural compounds against liver cancer. Sesquiterpenes, particularly alpha-bisabolol, have been shown to inhibit protective autophagy and promote apoptosis in diverse cancer models. In Chen’s study (154), alpha-bisabolol inhibited tumor cells in a dose- and time-dependent manner and showed the different cytotoxic effects on various human cancer cell lines. In particular, alpha-bisabolol seemed to have a stronger death effect on human liver cancer cell line HepG2. It might induce HepG2 apoptosis through first stimulating Fas-mediated apoptosis which led to elevated expression of Fas, then intrigued the activation of p53 and NF-κB. In addition, it is reported that alpha-bisabolol has the effect of anticancer through the mitochondrial pathway (155, 156). Intrinsic apoptosis mitochondrial mechanisms are effective in alpha-bisabolol-induced cell death, which is likely to mitochondrial and lysosomal death subroutines. The viability of cancer cells can be induced by alpha-bisabolol in a low-toxical way, which provides a perspective strategy of anticancer.

Autophagy is known for its dual roles in cancer development. On the one hand, autophagy can protect cells from dying under conditions, such as nutrient deprivation; on the other hand, autophagy can also lead to cell death (157). For example, PD exerts a cytoprotective mechanism in HrpG2 cells and participates in the formation of autophagosomes. Natural compounds can function in different stages of autophagy. Tetrandrine and oroxylin A affect the expression of *Atg-5* and *Atg-7*, which are two genes involved in the generation of LC3-II (158). Oroxylin A and berberine can upregulate Beclin-1 expression, thereby affecting the initiation process of autophagy. Allicin can decrease the cytoplasmic p53 levels, which induces autophagy and inhibits apoptosis. Tetrandrine acts *via* the ERK pathway, whereas oroxylin A, a multitarget compound, affects not only Beclin-1 but also the PI3K-PTEN-Akt-mTOR signalling pathway (**Table 3**).

**Figure 4:**
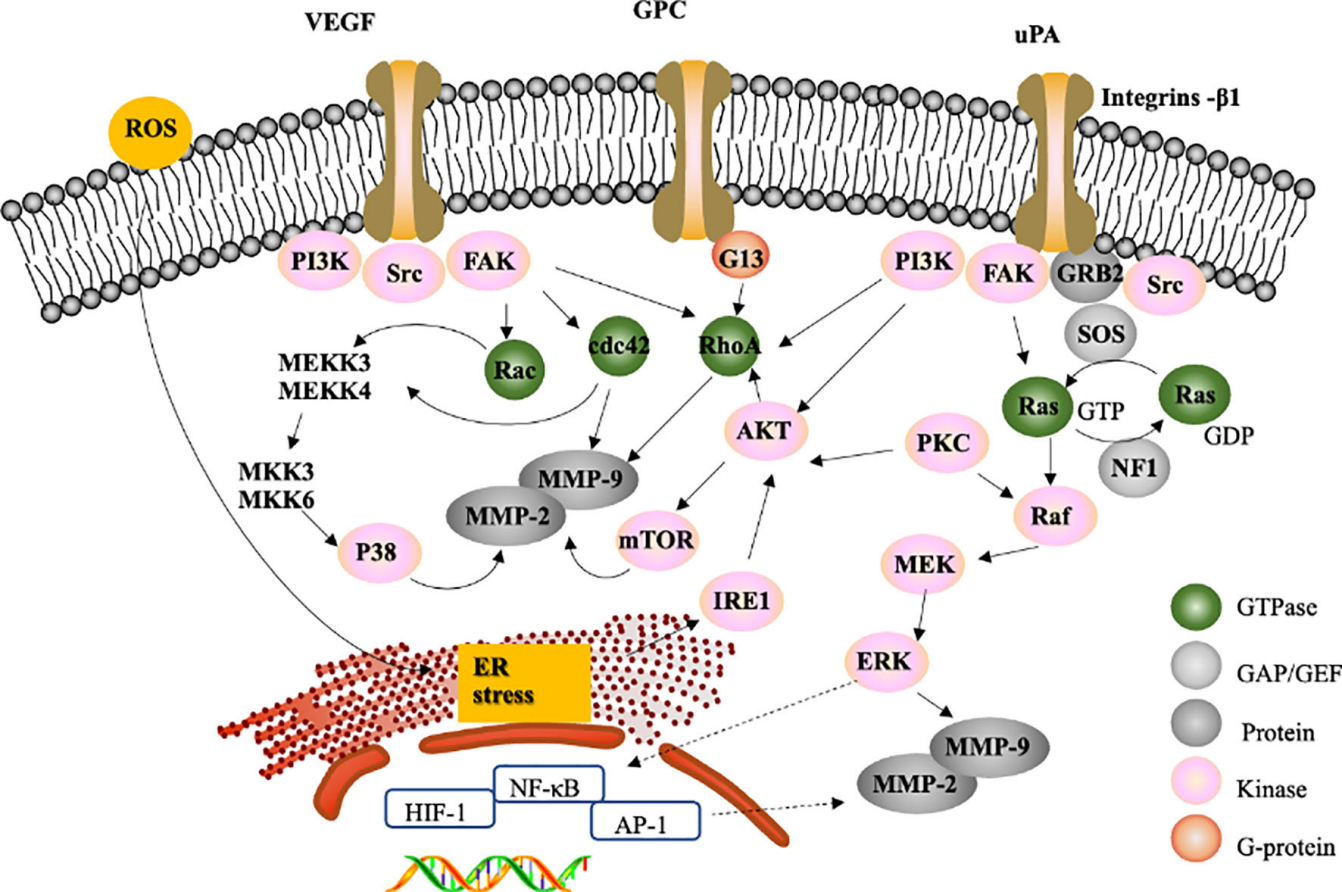
Proposed signalling pathway for the inhibition of migration and invasion. The effect of natural compounds likely occurs through the inhibition of FAK, a GTPase family that regulates MMP-2/-9 expression through PI3K/Ark pathways, the P38 MAPK pathway or the Ras/Raf/Erk signalling pathway to inhibit migration and invasion.

It has been reported that regulating the expression of MMP-2/-9 could inhibit the migration and invasion of HCC (124) (55). MMP-2 and MMP-9 are believed to be the key targets for inhibiting cell metastasis, and compounds could up- or downregulate MMP-2/-9 expression through various signalling pathways. Several natural compounds have been shown to inhibit HCC migration and invasion by modulating MMP-2/-9 expression and activity through the following three signalling pathways: Erk1/2, p38 MAPK and PI3K/Akt (**Figure 4**). In addition, RhoA is required for cell migration (150) and can abrogate the expression of MMP-9 to inhibit HCC metastasis (157) (**Figure 4**). Some natural compounds, such as 11-epi-sinullariolide acetate, isolated from *Sinularia flexibilis*, may inhibit metastasis through the ERK1/2, p38MAPK and FAK/PI3K/AKT/mTOR pathways to regulate the expression of MMP-2/-9.

TCMs are well known for their heterogeneous composition of molecules that affect multiple targets and pathways (158). Some natural compounds isolated from TCMs, such as hispolon, an active phenolic compound of *Phllinus linteus*, act on multiple targets. Hispolon has been reported to regulate the components of cell cycle progression and apoptotic machinery to delay the S to G2/M transition and induce apoptosis by increasing Bax expression and promoting cytochrome c release (55). Hispolon also inhibits cell migration and invasion *via* the inactivation of the Erk1/2 signalling pathway, RhoA protein expression and the PI3K pathway (100). Furthermore, MCL, a compound extracted from bitter gourd, targets caspase 8/9 to induce apoptosis and inhibits LC-II to trigger autophagy through a p53-independent pathway (75). These multifaceted compounds exhibit great potential in liver cancer therapy. Currently, sorafenib is the only drug used as the standard first-line treatment for advanced HCC, but drug resistance to sorafenib is a serious problem. Although much progress is needed before potential natural compounds are approved as clinical drugs for the treatment of HCC, some compounds, such as the ingredients of the TCMs Chan Su and bufalin, could improve sorafenib efficacy and reduce drug resistance. Scientists have investigated whether bufalin-mediated Akt activation through the IRE1 pathway to induce apoptosis can reverse resistance to sorafenib (131). This research paved the way for a new application of natural compounds as they may contribute to reducing the drug resistance of established treatments, such as sorafenib.

Targeted therapy with chemicals effectively prolongs the life of patients, while drug resistance hinders their application (159). Liver cancer stem cells are the key cells responsible for the occurrence and recurrence of liver cancer, and the key to curing tumors is to intervene with liver cancer stem cells (160). It can be pleasantly surprising to determine that multiple natural products play critical roles in terms of liver cancer recurrence and resistance to therapies. Luffa cylindrica gaffes, for example, can reduce the proportion of cancer stem cells in the blood of HCC patients, which reduces the recurrence and spread of cancer cells (161). Researchers found that its self-synthetic psyche base derivatives *in vitro* and *in vivo* can significantly inhibit the growth of human liver cancer cells and reduce the stem characteristics of liver cancer cells. This derivative may be achieved by suppressing the AKT/mTOR/p70S6K and AKT/GSK3 beta/β-catenin signalling pathways (162). Poplar is a flavonoid compound extracted from Chinese medicine wood butterflies. Poplar has a wide range of pharmacological activities (163). It has been found that poplar has an obvious inhibitory effect on the formation of the stem cell globulin formation rate of liver cancer and that poplar can reduce the protein expression of cell stem markers, blocking hedgehog signal transduction, thereby playing a role in inhibiting the development of liver cancer (164).

There are several risk factors for liver cancer, including hepatitis virus, alcohol addiction, liver cirrhosis and hepatotoxic carcinogens in moldy food (165). About 1/3 of patients with liver cancer have a history of chronic hepatitis may be related to chronic hepatitis B(HBV) and hepatitis C(HCV) (166, 167).Because of the high mortality of virus-caused HCC, antiviral therapy for HCV and HBV is urgently needed. Clinically HBV-induced HCC is usually characterized by elevated serum HBV-DNA levels and increases the risk of death in the advanced stages of cancer (168). The treatment of HBV is now done by vaccination, but even patients who have cured HBV are still likely to have HCC recurrence. Therefore, it is more urgent to find feasible ways to treat viral hepatitis thoroughly.

TCM has become a popular area of research in recent years. As natural compounds attract significant attention from the scientific community and the pharmacological effects and their potential mechanisms against hepatocellular carcinoma are well studied in laboratory work, clinical data are needed to confirm the specific efficacy of these compounds. With their advantages of targeting multiple effectors and pathways, many anticancer natural compounds, such as curcumin and resveratrol, are currently undergoing clinical trials (169, 170). Based on the database of several clinical studies investigating curcumin, curcumin improves the effectiveness of chemotherapy and radiotherapy. The expression of antimetastatic proteins was also increased along with reduced side effects (171). Combination therapy may be a good choice for natural compounds in clinical applications. For resveratrol, based on available clinical results, subsequent clinical trials are currently investigating dose limits to address resveratrol’s poor bioavailability with regard to extrapolating its effects to humans (172). In addition, clinical research investigating valuable Chinese herbal medicine is also ongoing. A recent randomized, double-blind, and placebo-controlled clinical trial investigating Ejiao (Asini Corii Colla) showed a promising effect in women suffering from blood deficiency (173). To apply natural medicines in clinical settings, the factors influencing the action of drugs, such as delivery systems, formulations, modulation of metabolism, and interactions with other compounds, should be considered. In contrast, being a “multitarget” drug may lead to lower selectivity, thereby increasing the incidence of adverse effects. Elucidating the exact mechanisms of these compounds and analysing their clinical efficacy still requires further work. As illustrated in the success of artemisinin in the treatment of malaria, natural compounds isolated from TCMs may hold the key to combating other disease entities, including cancer.

In this review, the pathways and targets of HCC were described in detail to help researchers propose new strategies to seek selected natural products for the treatment of HCC by the regulation of different targets. Due to the high incidence and mortality of liver cancer and because treatment with chemical drugs causes physical damage to varying degrees, it is particularly important to extract low-toxicity and effective natural compounds from traditional Chinese medicine.

## Author Contributions

HX and MY proposed the conception and design. YZ and WZ performed the data collection and drafted the manuscript. LX generated the figures and tables. HZ, MY, and HX reviewed the data and provided final approval of the manuscript. All authors contributed to the article and approved the submitted version.

## Conflict of Interest

The authors declare that the research was conducted in the absence of any commercial or financial relationships that could be construed as a potential conflict of interest.

## Publisher’s Note

All claims expressed in this article are solely those of the authors and do not necessarily represent those of their affiliated organizations, or those of the publisher, the editors and the reviewers. Any product that may be evaluated in this article, or claim that may be made by its manufacturer, is not guaranteed or endorsed by the publisher.
